# Strength, Carbonation Resistance, and Chloride-Ion Penetrability of Cement Mortars Containing Catechol-Functionalized Chitosan Polymer

**DOI:** 10.3390/ma14216395

**Published:** 2021-10-25

**Authors:** Se-Jin Choi, Sung-Ho Bae, Jae-In Lee, Eun-Ji Bang, Haye-Min Ko

**Affiliations:** 1 Department of Architectural Engineering, Wonkwang University, Iksan 54538, Korea; csj2378@wku.ac.kr (S.-J.C.); caos1344@naver.com (S.-H.B.); wodls103@naver.com (J.-I.L.); 2 Department of Chemistry, Wonkwang University, Iksan 54538, Korea; dmswlal1@naver.com; 3 Department of Chemistry, Wonkwang Institute of Material Science and Technology, Wonkwang University, Iksan 54538, Korea

**Keywords:** Cat-Chit polymer, cement mortar, compressive strength, carbonation depth, chloride-ion penetrability

## Abstract

There have been numerous recent studies on improving the mechanical properties and durability of cement composites by mixing them with functional polymers. However, research into applying modified biopolymer such as catechol-functionalized chitosan to cement mortar or concrete is rare to the best of our knowledge. In this study, catechol-functionalized chitosan (Cat-Chit), a well-known bioinspired polymer that imitates the basic structures and functions of living organisms and biological materials in nature, was synthesized and combined with cement mortar in various proportions. The compressive strength, tensile strength, drying shrinkage, accelerated carbonation depth, and chloride-ion penetrability of these mixes were then evaluated. In the ultraviolet–visible spectra, a maximum absorption peak appeared at 280 nm, corresponding to catechol conjugation. The sample containing 7.5% Cat-Chit polymer in water (CPW) exhibited the highest compressive strength, and its 28-day compressive strength was ~20.2% higher than that of a control sample with no added polymer. The tensile strength of the samples containing 5% or more CPW was ~2.3–11.5% higher than that of the control sample. Additionally, all the Cat-Chit polymer mixtures exhibited lower carbonation depths than compared to the control sample. The total charge passing through the samples decreased as the amount of CPW increased. Thus, incorporating this polymer effectively improved the mechanical properties, carbonation resistance, and chloride-ion penetration resistance of cement mortar.

## 1. Introduction

Concrete is a widely used material in the construction field for its many advantages, such as its low cost, excellent compressive strength, and high durability [1]. However, its tensile strength is low, and it is vulnerable to cracks due to various causes, such as shrinkage or chemical reactions. In addition, maintenance activities, such as repairing cracks, can be significantly costly [2]. Over the past decade, numerous studies have developed various functional admixtures (shrinkage-reducing agents, watertight admixtures, etc.), fiber reinforcements (e.g., Polyvinyl alcohol (PVA) fiber and amorphous metallic fiber), and polymer materials to overcome these problems in cement mortar or concrete [3,4,5,6,7,8,9,10].

Recently, various research studies have mixed functional polymers into cement composites in order to enhance the mechanical properties and durability of cement composites [11,12,13,14,15,16,17,18,19]. Jo et al. [11] examined the characteristics of polymer concrete using unsaturated polyester resins from recycled polyethylene terephthalate plastic waste and recycled concrete aggregate; they reported that the strength of polymer concrete increased as the resin content increased. Ferdous et al. [12] investigated the effect of the resin-to-filler ratio on the mechanical properties of epoxy-based polymer concrete, and they found that the resin-to-filler ratio significantly affects the spatial distribution of aggregates. Meanwhile, Azadmanesh et al. [13] used a polymer of styrene butadiene rubber and ethylene vinyl acetate to improve the mechanical properties of Engineered Cementitious Composites (ECC) using unoiled fibers. Their findings showed that the use of polymers can significantly improve the tensile and flexural strength properties of ECC. Mohesson et al. [14] characterized concrete mixed with alginate (a biopolymer), which increased both the compressive and tensile strengths of the concrete. In addition, Shanmugavel et al. [15] reported that adding biopolymers from cactus extract to cement paste increased both the viscosity and durability of the concrete.

However, in general, most studies employed conventional resin, epoxy, etc., as polymer additives in cement composites, but no studies have specifically focused on applying modified bioinspired polymers to cement mortar or concrete. 

In this study, catechol-functionalized chitosan (Cat-Chit), a well-known bioinspired polymer that imitates the basic structures and functions of living organisms and biological materials in nature, was synthesized to examine its applicability in cement mortar. Chitosan, the most popular biopolymer, is obtained by the deacetylation of chitin, which is widely found in crustaceans. In recent decades, the applications of Cat-Chit in biomedicine have been well established, such as tissue adhesives [20] and drug delivery systems [21], because of its medical benefits as well as its low toxicity and biodegradability. However, its low solubility in aqueous solutions hindered its further advancement in this field [22,23,24,25,26,27,28]. In order to overcome this critical issue, chitosan-based derivatives have been synthesized via amide formation with 3,4-dihydroxyhydrocinnamic acid and amine [20,21,29,30,31,32]. The catechol group in the chitosan backbone increases not only its solubility but also its adhesion to tissues. Cat-Chit polymers (CCPs) have also been modified by using several nanoparticles [33,34]. These results have attracted significant attention to CCPs owing to its superior capability as a versatile material.

Therefore, in this study, we synthesized a catechol-functionalized chitosan polymer (CCP) and examined its applicability to cement mortar. To this end, we analyzed this CCP, and we evaluated mortar samples containing CCP in terms of their compressive strength, tensile strength, drying shrinkage, accelerated carbonation depth, and chloride-ion penetrability.

## 2. Materials and Experimental Methods 

### 2.1. Materials

In order to synthesize CCP, Chitosan (medium molecular weight; degree of deacetylation: 75–85%) and hydrocaffeic acid (HCA; 3-(3,4-dihydroxyphenyl)propionic acid) [35] were used in this study. In addition, we used 1-Ethyl-3-(3-dimethylaminopropyl)carbodiimide hydrochloride (EDC) and ethanol. All the chemicals were of analytical grade and were used without further purification. A Q-Grad 1 purification cartridge from Millipore water purification systems was used to obtain ultrapure water. 

**Figure 1:**
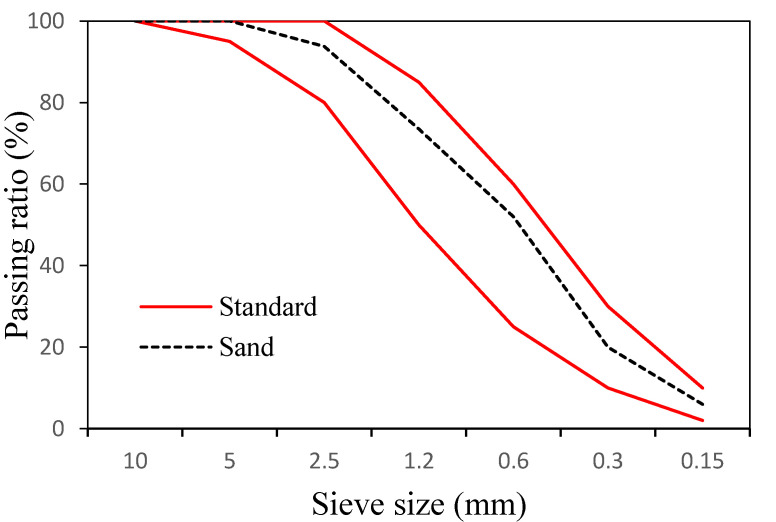
Particle size distribution of fine aggregates used in this study.

In addition, we used ASTM typeⅠordinary Portland cement with a specific gravity of 3.15 made by Asia Co. (Seoul, Korea). Table 1 lists its chemical composition. For fine aggregates, sand from the Namwon region with a specific gravity of 2.6 and a fineness modulus of 2.89 was used. Figure 1 shows the particle size distribution of the fine aggregate.

### 2.2. Mix Proportions and Specimen Preparation

Table 2 lists the mix proportions used in this study. The water–cement ratio (W/C) was fixed at 50%, which is widely used for conventional concrete. A CCP solution, in which 500 mg of CCP was dissolved in 1000 mL of water (hereinafter, CPW), was mixed at proportions of 0%, 2.5%, 5.0%, 7.5%, and 10.0% of mixing water. For the sake of simplicity, samples are labeled with a suffix indicating the amount of CPW, e.g., CPW2.5 contained 2.5% CPW, as shown in Table 2. These CPW dosages correspond to 1.25% (CPW2.5) and to 5.0% (CPW10) of the cement weight.

A mechanical mixer was used to mix the cement mortar. Then, cubic specimens with dimensions 50 mm × 50 mm × 50 mm were prepared via molding for compressive strength testing, and cylindrical specimens of dimensions ∅50 mm × 100 mm were prepared for split-tensile strength testing. Additionally, specimens of dimensions 40 mm × 40 mm × 160 mm were prepared for drying shrinkage and accelerated carbonation testing, and cylindrical specimens of dimensions of ∅100 mm × 50 mm were prepared for the chloride ion penetration test.

Each specimen was demolded after 24 h and cured in water at 20 °C. The flow and compressive strength of the cement mortar mixes were measured according to the standard KS L 5105 [36], and the tensile strength was measured according to KS F 2423 [37]. The drying shrinkage of the samples was measured according to the standard KS F 2424 [38]. In the accelerated carbonation test, the carbonation depth was measured by using a phenolphthalein solution after the carbonation process in an accelerated carbonation chamber according to the standard KS F 2584 [39]. Additionally, the chloride-ion penetration test was performed according to ASTM C 1202 [40]. This test method involves monitoring the amount of electrical current passed through the samples. The total charge passed, in coulombs, has been found to be related to the resistance of the specimen to chloride ion penetration [40].

## 3. Experimental Results and Discussion

### 3.1. CCP Synthesis

**Figure 2:**
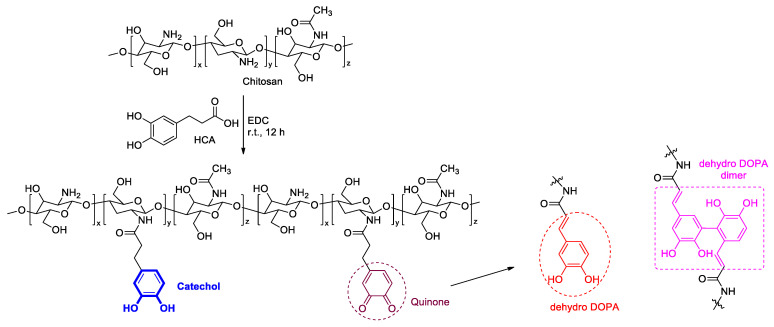
Synthesis of Cat-Chit and possible cross-linking pathways of catechol.

CCP was synthesized using EDC and HCA with chitosan, as previously reported (Figure 2) [35,41]. First, chitosan (500 mg) was dissolved in a 5 N HCl solution (2.5 mL) and DDW (23 mL). Then, HCA (590 mg) in ethanol (2.5 mL) and EDC (625 mg) in ethanol (10 mL) were slowly added to the chitosan solution. The pH of the resultant solution was adjusted to 5.0 by using a saturated NaOH solution, and the reaction mixture was stirred at room temperature for 12 h. Next, the pH of the mixture was again adjusted to 5.0 using the 5 N HCl solution. The residue was dialyzed using a membrane against a 10 mM NaCl solution for two days and DDW for 24 h, with a change of dialysate every 6 h. Finally, the solution was frozen at −20 °C in a refrigerator and lyophilized. 

In addition, the absorbance of the Cat-Chit solution in DDW was analyzed by using a Genesys 180 ultraviolet–visible (UV−vis) spectrophotometer (Thermo Fisher, Waltham, MA, USA). Moreover, in order to conduct nuclear magnetic resonance (NMR) spectroscopy, Cat-Chit was dissolved in D_2_O. Then, ^1^H-NMR spectra were recorded using a 500 MHz NMR spectrometer (JEOL).

In order to investigate the interaction of Cat-Chit with a basic adduct from cement mortar, 40 mg of Cat-Chit reacted with a Ca(OH)_2_ solution (296 mg/40 mL; this corresponds to a 0.1 M concentration, which creates a similar environment to the adducts that form in cement mortar) at room temperature for 12 h, after which the solid residue was washed with water and lyophilized, yielding a brown solid. The interaction between calcium and CCP was analyzed by Fourier transform infrared (FT-IR) spectroscopy using this solid. A mid-infrared (400–4000 cm^–1^) range Nicolet iS5 FT-IR Spectrometer (Thermo Scientific, Waltham, MA, USA) was used to collect spectra in the transmittance band between 400 and 4000 cm^–1^ at a resolution of 1 cm^–1^. In addition, scanning electron microscopy (SEM) images were recorded using an AIS1800C (SERON Technologies, Seoul, Korea).

### 3.2. CCP Characterization

**Figure 3:**
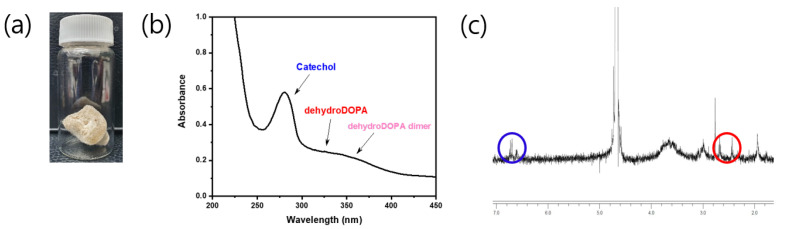
Qualitative analysis of Cat-Chit: (**a**) photograph of the product; (**b**) UV–vis spectrum; (**c**) ^1^H-NMR.

The catechol group of the CCP was confirmed by UV–vis and ^1^H NMR spectroscopies (Figure 3b,c). The UV–vis spectra revealed a maximum absorption peak of 280 nm, which corresponded to the catechol conjugation; in addition, aromatic protons of catechol were represented at approximately 6.87 ppm in the ^1^H NMR spectra. Remarkably, the CCP was partially oxidized to quinone from catechol, as catechol is prone to oxidation. This quinone, a well-known and highly reactive species, was converted to 3,4-dihydroxyphenylalanine (dehydro-DOPA) moieties or dehydro-DOPA dimers (Figure 2) [42,43,44]. The UV–vis spectra of the synthesized CCP revealed a peak at approximately 330 nm and a broad peak at 350 nm, which corresponded to the dehydro-DOPA [44] and the dehydro-DOPA dimer [45], respectively. Additionally, although catechol showed one multiple-type peak, quinone exhibited two peaks between 6.5 and 7.0 ppm (Figure 3c, blue circle). A weak, broad peak at approximately 2.5 ppm was attributed to the methylene protons of HCA, whereas the protons of quinone appeared as two separate weak peaks (Figure 3c, red circle). Changes in the structure of the synthesized CCP were reflected by the light brown color of the product (Figure 3a), and the decrease in the solubility relative to that of Cat-Chit was observed.

**Figure 4:**
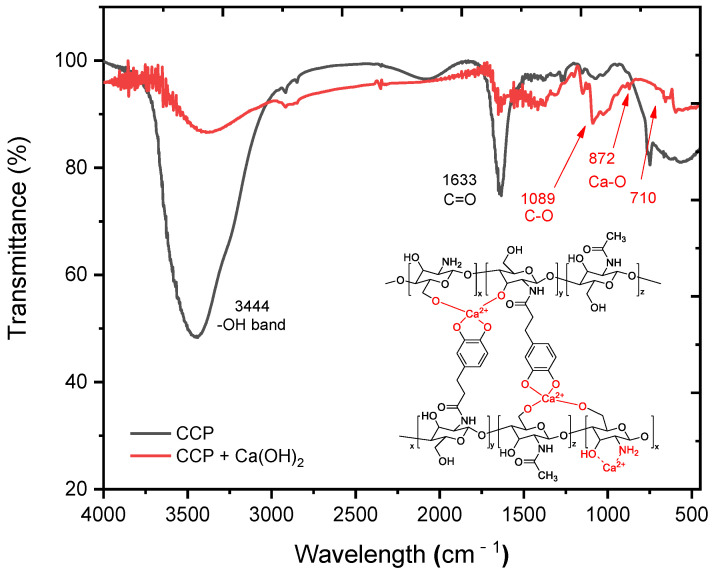
FT-IR spectra of CCP (black line) and CCP upon reacting with Ca(OH)_2_ (red line). The expected structure of calcium–Cat-Chit was added in the spectra.

In order to understand the interaction mechanism of the CCP with cement mortar, changes in the functional groups of the CCP were compared by using FT-IR before and after allowing the CCP to react with Ca(OH)_2_ (Figure 4). Before the reaction, the peak at approximately 3444 cm^–1^ corresponded to the hydroxy and amino groups in the CCP, and another peak at approximately 1633 cm^–1^ corresponded to carbonyl of amide (Figure 4 (black line)). In contrast, after treating CCP with Ca(OH)_2_, the absorption peak at approximately 3444 cm^–1^ was considerably attenuated because of the reaction with hydroxy and hydroxide, but significant new peaks appeared at 872 and 710 cm^–1^, representing the Ca–O bonds (Figure 4 (red line)). This observation strongly suggests that an interaction occurred between CCP and Ca(OH)_2_, such as a cross-linking or chelation process. 

**Figure 5:**
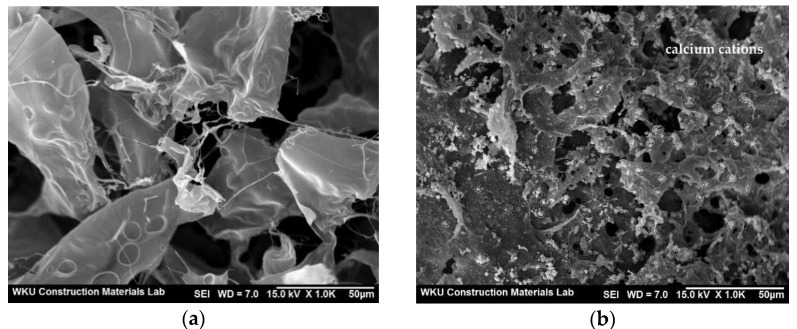
SEM images showing the morphology of (**a**) CCP and (**b**) calcium–CCP.

The SEM images in Figure 5a,b reveal the morphology of the CCP and calcium–CCP, respectively. In contrast to the transparent, smooth surface of the CCP, the calcium cations in calcium–CCP were clearly detected in the form of spherical mineral deposits, and the rest of the surface appeared strong and rigid.

### 3.3. Mortar Flow

**Figure 6:**
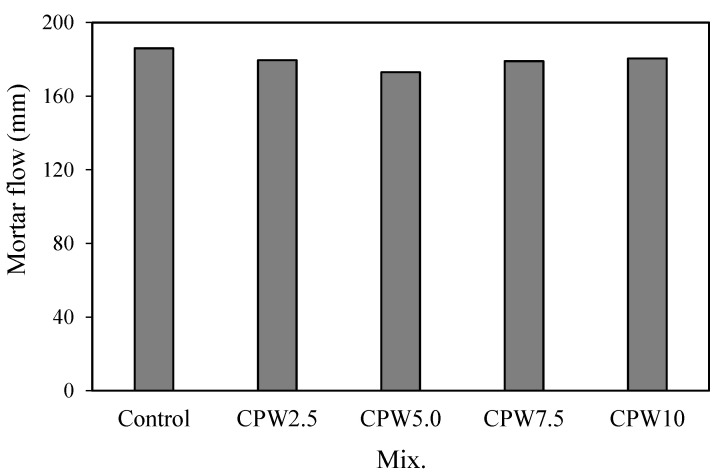
Mortar flow of the samples.

Figure 6 shows the variations in the flow of mortars with various amounts of CPW. Evidently, the mortar flow of the control mix was the highest at approximately 186 mm, whereas those of the mixtures mixed with CPW were all slightly lower in the range of approximately 173–181 mm. However, these differences in the flow were not significant, implying that the incorporation of the CCP did not significantly degrade the fluidity of the cement mortar.

### 3.4. Compressive Strength

**Figure 7:**
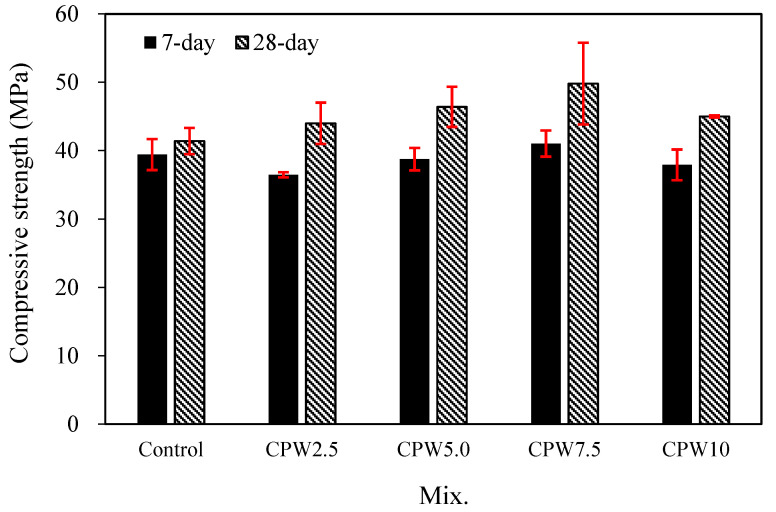
Compressive strength of the samples after 7 and 28 days.

Figure 7 shows the changes in the compressive strengths of the mortars with various amounts of CPW after 7 and 28 days. After 7 days, the compressive strength of the control sample with no CPW was found to be approximately 39.4 MPa. By contrast, the 7-day compressive strength of the CPW7.5 sample was approximately 41.0 MPa, which was higher than that of other mixes and approximately 4% higher than that of the control sample.

This trend was more pronounced after 28 days. Specifically, while the 28-day compressive strength of the control sample was approximately 41.4 MPa, those of all the CPW samples were in the range of 44.0–49.8 MPa, indicating that they all had relatively higher compressive strengths than the control sample. In particular, the CPW7.5 sample again exhibited the highest compressive strength, viz., 49.8 MPa after 28 days, which is approximately 20.2% higher than that of the control sample.

According to previous investigations [46,47,48] and our own study of the interaction mechanism, hydroxyl or amino groups of the CCP seem to participate in cross-linking or chelation reactions under certain basic conditions, such as the presence of Ca(OH)_2_ solution. These processes could be promoted to make the polymer more rigid through interactions among many functional groups in a high-concentration polymer solution. Indeed, the experimental results showed that the system with calcium–CCP cross-linking or chelating complexes had a higher compressive strength than the control system with no CPW. 

**Figure 8:**
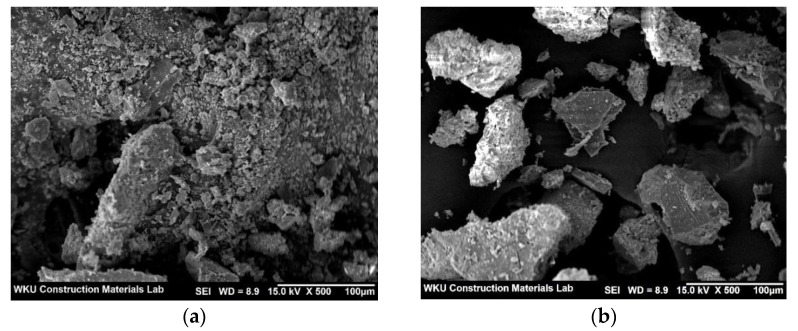
SEM images of the (**a**) control and (**b**) CPW7.5 samples.

Figure 8a,b show SEM images of the control sample and CPW7.5, respectively, after the 28-day compressive strength test. As shown in the figure, the surface of the CPW7.5 particles appears to be denser than that of the control sample.

In addition, in the samples containing 2.5–7.5% of CPW, the compressive strength increased with increasing CPW proportion, but in the CPW10 sample, the compressive strength decreased relative to that of the CPW7.5 sample. This degradation was probably caused by self-aggregation [49], which generally occurs at higher polymer concentrations and is expected to decrease compressive strength. Therefore, mixing an appropriate amount of CCP with cement mortar was found to effectively improve the mechanical properties of the cement mortar. 

### 3.5. Tensile Strength

**Figure 9:**
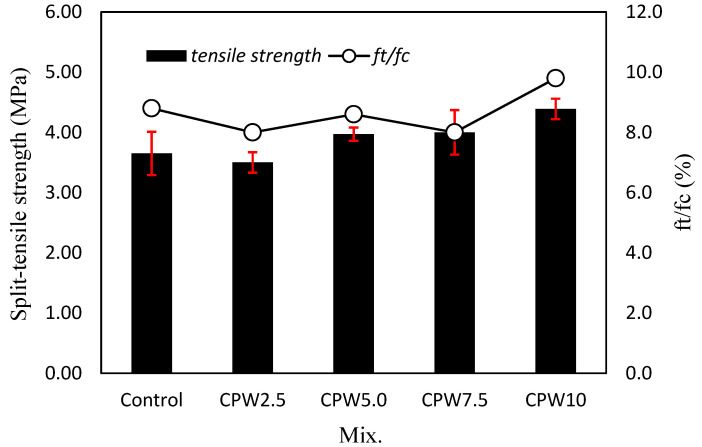
Tensile strength (left axis) and ratio of the tensile strength to compressive strength (right axis) of various mixed samples.

Figure 9 shows the 28-day tensile strength of the mortar according to the amount of CPW. Evidently, the tensile strength of the mortar tends to increase as the CPW ratio increases. Specifically, the tensile strength of the samples containing 5% or more CPW (i.e., CPW5.0, CPW7.5, and CPW10) was approximately 3.97–4.39 MPa, which was ~2.3–11.5% higher than that of the control sample. In addition, the tensile strength of CPW10 sample was higher than that of the CPW7.5 sample, indicating that the use of more CCP effectively increased the tensile strength of mortar. Therefore, the incorporation of CCP improves both the tensile and compressive strength of the mortar. In addition, the ratio of the tensile strength (ft) to compressive strength (fc) was approximately 8.0–9.8%, which was similar regardless of the amount of CPW.

### 3.6. Drying Shrinkage

**Figure 10:**
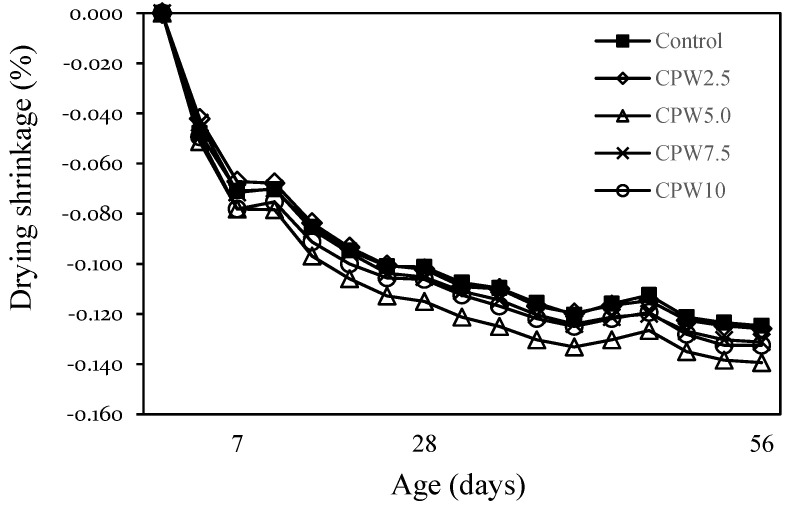
Drying shrinkage of samples.

Figure 10 shows the change in the drying shrinkage of mortar after 56 days according to the amount of CPW. The drying shrinkages of the control sample and CPW2.5 were approximately 0.125–0.126% after 56 days, whereas those of CPW7.5 and CPW10 were also similar (~0.131–0.133%). However, the drying shrinkage of CPW5.0 was approximately 0.139%, which was ~11% higher than that of the control sample. In general, various factors affect the drying shrinkage of the mortar and concrete. In this study, the drying shrinkage of CPW5.0 was relatively large, but it is not clear whether this difference could be attributed to the presence of CCP or any other influencing factors. More detailed studies on shrinkage, including drying shrinkage and autogenous shrinkage of cement composites by the CCP proportion, are thus required.

### 3.7. Accelerated Carbonation Depth

**Figure 11:**
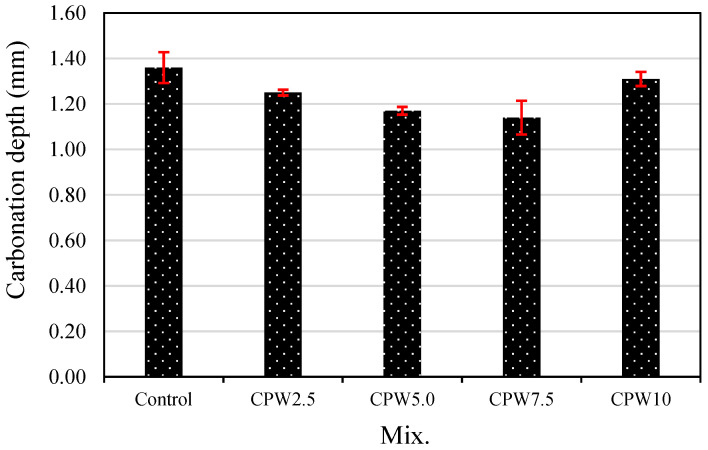
Accelerated carbonation depth of the samples.

Figure 11 shows the carbonation depth after 28 days of accelerated aging of the various samples. The carbonation depth of the control sample was approximately 1.36 mm, which was higher than those of all the mixes containing CCP. In the case of samples containing 0–7.5% CCP, carbonation depth decreased as the CPW proportion increased. Particularly, the carbonation depth of CPW7.5 was approximately 1.14 mm, which was about 16% lower than that of the control sample and the lowest among all the samples. Therefore, the proper incorporation of CCP effectively improves not only mechanical properties, such as the compressive and tensile strength (Figure 8 and Figure 9, respectively), but also carbonation resistance. Taken together, these findings suggest that adding CPW makes the cement composites denser, which is known to increase compressive strength and decrease carbonation depth. 

### 3.8. Chloride-Ion Penetrability

**Figure 12:**
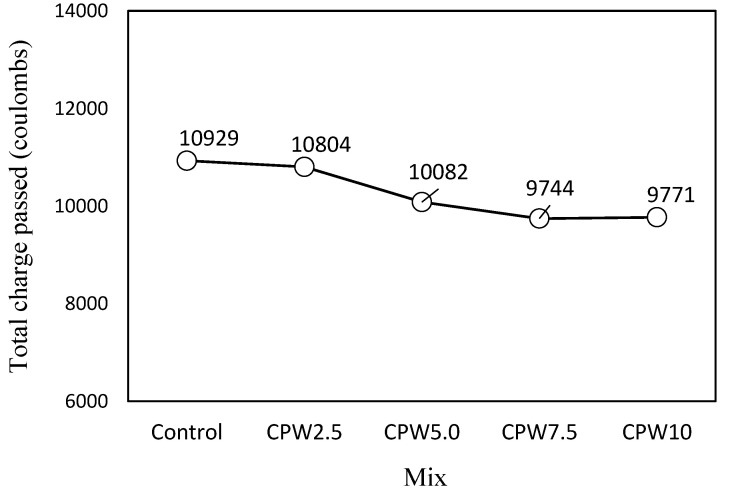
Chloride-ion penetrability of the samples after 28 days.

As shown in Figure 12, which illustrates the chloride-ion penetrability according to the amount of CPW, the total charge passing through the control mixture was the highest at approximately 10929 C. For the CPW2.5 sample, this value was slightly lower, and it continued to decrease with an increasing amount of CPW up to CPW7.5, which had a similar value to CPW10, (9744 and 9771 C, respectively). Therefore, the use of CCP is expected to improve the resistance of cement mortar relative to chloride-ion penetration.

## 4. Conclusions

This paper describes the first application of CCP to cement mortar in order to improve its mechanical properties and durability. The following conclusions can be derived from this study.

The results showed that an interaction between the synthesized polymer and Ca(OH)_2_ made CCP compatible with cement mortars. 

The flow of samples with and without the CCP was not significantly different, implying that the incorporation of the CCP did not significantly affect the fluidity of the cement mortar.

The CPW7.5 sample exhibited the highest 7-day and 28-day compressive strength. Specifically, after 28 days, its compressive strength was 49.8 MPa, which was approximately 20.2% higher than that of the control sample. The tensile strength of the CPW5.0, CPW7.5, and CPW10 samples was approximately 3.97–4.39 MPa, which was ~2.3–11.5% higher than that of the control sample.

The carbonation depth of CPW7.5 was approximately 1.14 mm, which was about 16% lower than that of the control sample. Therefore, the proper incorporation of CCP effectively improves not only mechanical properties but also carbonation resistance.

The total charge passing through the samples decreased as the amount of CPW increased. Therefore, CCP is concluded to improve chloride ion penetration resistance of cement mortar.

Further studies are required in order to establish how the presence of CCP affects viscosity, shrinkage, and long-term mechanical strength, as well as how CCP influences the relationship between the microstructures of cement composites and their mechanical properties and durability characteristics.

## Figures and Tables

**Table 1:** Chemical composition of the cement used in this study.

Components	SiO_2_	Al_2_O_3_	Fe_2_O_3_	CaO	MgO	K_2_O
Ratio (%)	17.43	6.50	3.57	64.40	2.55	1.17

**Table 2:** Samples and their mix proportions used in this study.

Mix	CPW (%)	W/C (%)	Water (kg/m^3^)	Cement (kg/m^3^)	Sand (kg/m^3^)
Control CPW2.5 CPW5.0 CPW7.5 CPW10	0 2.5 5.0 7.5 10	50	170	340	739

## Data Availability

Not applicable.
